# Unveiling Gestational Diabetes: An Overview of Pathophysiology and Management

**DOI:** 10.3390/ijms26052320

**Published:** 2025-03-05

**Authors:** Rahul Mittal, Karan Prasad, Joana R. N. Lemos, Giuliana Arevalo, Khemraj Hirani

**Affiliations:** Diabetes Research Institute, Miller School of Medicine, University of Miami, Miami, FL 33136, USA; knp58@miami.edu (K.P.); joanalemos@miami.edu (J.R.N.L.); gxa677@med.miami.edu (G.A.)

**Keywords:** gestational diabetes mellitus, glucose intolerance, maternal health, neonatal outcomes, hyperglycemia, preeclampsia, insulin resistance, dietary intervention

## Abstract

Gestational diabetes mellitus (GDM) is characterized by an inadequate pancreatic β-cell response to pregnancy-induced insulin resistance, resulting in hyperglycemia. The pathophysiology involves reduced incretin hormone secretion and signaling, specifically decreased glucagon-like peptide-1 (GLP-1) and glucose-dependent insulinotropic polypeptide (GIP), impairing insulinotropic effects. Pro-inflammatory cytokines, including tumor necrosis factor-alpha (TNF-α) and interleukin-6 (IL-6), impair insulin receptor substrate-1 (IRS-1) phosphorylation, disrupting insulin-mediated glucose uptake. β-cell dysfunction in GDM is associated with decreased pancreatic duodenal homeobox 1 (PDX1) expression, increased endoplasmic reticulum stress markers (CHOP, GRP78), and mitochondrial dysfunction leading to impaired ATP production and reduced glucose-stimulated insulin secretion. Excessive gestational weight gain exacerbates insulin resistance through hyperleptinemia, which downregulates insulin receptor expression via JAK/STAT signaling. Additionally, hypoadiponectinemia decreases AMP-activated protein kinase (AMPK) activation in skeletal muscle, impairing GLUT4 translocation. Placental hormones such as human placental lactogen (hPL) induce lipolysis, increasing circulating free fatty acids which activate protein kinase C, inhibiting insulin signaling. Placental 11β-hydroxysteroid dehydrogenase type 1 (11β-HSD1) overactivity elevates cortisol levels, which activate glucocorticoid receptors to further reduce insulin sensitivity. GDM diagnostic thresholds (≥92 mg/dL fasting, ≥153 mg/dL post-load) are lower than type 2 diabetes to prevent fetal hyperinsulinemia and macrosomia. Management strategies focus on lifestyle modifications, including dietary carbohydrate restriction and exercise. Pharmacological interventions, such as insulin or metformin, aim to restore AMPK signaling and reduce hepatic glucose output. Emerging therapies, such as glucagon-like peptide-1 receptor (GLP-1R) agonists, show potential in improving glycemic control and reducing inflammation. A mechanistic understanding of GDM pathophysiology is essential for developing targeted therapeutic strategies to prevent both adverse pregnancy outcomes and the progression to overt diabetes in affected women.

## 1. Introduction

**Figure 1:**
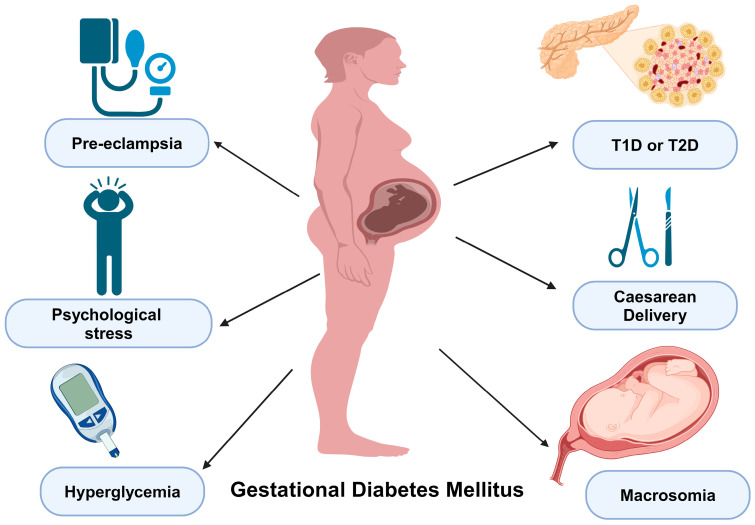
Outcomes of gestational diabetes mellitus (GDM) on mothers and offspring: This figure illustrates short-term and long-term outcomes of GDM for both mothers and their offspring. For mothers, short-term outcomes include increased risk of cesarean delivery and hypertensive disorders of pregnancy, while long-term outcomes encompass a heightened risk of developing type 1 diabetes (T1D) or type 2 diabetes (T2D). For offspring, short-term outcomes highlight the risks of macrosomia, neonatal hypoglycemia, and respiratory distress syndrome. Long-term outcomes for offspring demonstrate an increased risk of T1D or T2D and metabolic syndrome into adulthood. Created in BioRender. https://BioRender.com/n76l932 (accessed on 2 December 2024).

Gestational diabetes mellitus (GDM) is a multifaceted metabolic disorder characterized by increased blood sugar levels during pregnancy [1,2,3,4,5,6,7,8,9,10,11,12,13]. It poses a substantial healthcare challenge during gestation, influencing the well-being of both the mother and the developing fetus (Figure 1) [6,7,8,9,10]. GDM is generally defined as any degree of glucose intolerance with onset or first recognition during pregnancy [1]. Typically emerging in the second and third trimester, GDM is associated with pancreatic *β*-cell dysfunction or delayed response to glucose levels and substantial insulin resistance second to placental hormonal release [2,3,4,5,6,7,8,9,10,11,12,13]. The global prevalence of GDM has been on the rise, largely attributed to factors such as increased maternal age, obesity, and sedentary lifestyles [14,15,16,17]. However, the exact prevalence varies widely depending on the population studied, diagnostic criteria used, and geographic region. In 2021, a study reported the global prevalence of GDM as 14.7%, based on the International Association of Diabetes and Pregnancy Study Groups (IADPSG) criteria, the most used screening method worldwide. In 2023, the Centers for Disease Control and Prevention (CDC) reported that the proportion of mothers diagnosed with GDM increased from 6.0% in 2016 to 8.3% by 2021 [18]. This upward trend in GDM diagnosis was observed across all maternal age groups. Furthermore, a clear correlation between increasing maternal age and higher rates of GDM was evident. The CDC also found that in 2021 the incidence of GDM in mothers aged 40 years and older reached 15.6%, which was nearly six-fold the rate observed in mothers under 20 years, who had a rate of just 2.7% [18]. This data highlights the increasing concern of GDM as a significant health issue, affecting a broader demographic and more pronouncedly impacting older pregnant women.

The rising prevalence of GDM can be attributed to a combination of demographic, lifestyle, and healthcare factors [19]. The key contributors include the increasing prevalence of obesity, metabolic syndrome, and sedentary lifestyles, all of which are well-established risk factors for GDM [20,21,22,23]. Furthermore, the trend toward delayed childbearing, resulting in a greater proportion of pregnancies occurring in women of advanced maternal age, significantly elevates the risk of developing GDM due to age-associated insulin resistance and β-cell dysfunction [24,25,26,27]. Advances in diagnostic practices, including more stringent screening criteria and widespread adoption of universal screening protocols, have also led to improved case detection, thereby contributing to the observed rise in prevalence. Genetic and epigenetic predispositions, coupled with disparities in socioeconomic conditions and healthcare access, exacerbate the burden of GDM, particularly in certain ethnic and racial groups with higher susceptibility [28,29,30,31,32]. Collectively, these factors highlight the urgent need for multifaceted strategies, including public health initiatives aimed at obesity prevention, early risk assessment, and equitable access to high-quality prenatal care, to address the escalating incidence of GDM and its associated maternal and fetal complications.

GDM is a metabolic disorder with profound implications for both maternal and fetal health (Figure 1) [33]. This condition is associated with multiple complications, including preeclampsia, hyperglycemia, increased risk of cesarean delivery, psychological stress, and the potential progression to type 1 or type 2 diabetes (Figure 1) [34,35,36]. Additionally, excessive fetal growth, or macrosomia, is a significant consequence of maternal hyperglycemia (Figure 1). Each of these outcomes has been correlated with complex metabolic, hormonal, and immunological pathways that interact dynamically throughout pregnancy [1].

Preeclampsia, a common comorbidity of GDM, is characterized by endothelial dysfunction and systemic inflammation, often exacerbated by dysregulated immune signaling [37,38]. Pro-inflammatory cytokines such as tumor necrosis factor-alpha (TNF-α) and interleukin-6 (IL-6) contribute to insulin resistance and vascular dysfunction, further complicating glucose metabolism [39]. The persistence of inflammatory mediators postpartum may also explain why women with a history of GDM have an elevated risk of developing chronic hypertension and cardiovascular disease later in life [40,41]. Similarly, autoimmune factors play a role in the progression from GDM to type 1 diabetes (T1D) in a subset of women, where the presence of autoantibodies against pancreatic islet cells can indicate an impending β-cell decline, leading to insulin dependence [42].

Psychological stress is a significant contributor to GDM, primarily through the activation of the hypothalamic–pituitary–adrenal (HPA) axis [43,44]. Chronic stress induces sustained hypercortisolemia, which impairs insulin sensitivity by promoting hepatic gluconeogenesis and inhibiting peripheral glucose uptake [45]. Additionally, elevated catecholamine secretion exacerbates insulin resistance by augmenting lipolysis and free fatty acid mobilization, further disrupting glycemic homeostasis [46,47]. The bidirectional relationship between psychological stress and metabolic dysfunction underscores the necessity of integrating stress management strategies into GDM management to optimize maternal–fetal outcomes [48].

Concomitantly, GDM itself imposes significant psychological distress on affected individuals, contributing to elevated anxiety and emotional dysregulation throughout gestation [49]. The diagnosis of GDM often precipitates heightened psychological burden, characterized by fear, guilt, and concern regarding adverse maternal and neonatal outcomes [50,51]. Stringent glycemic management, necessitating dietary modifications, frequent glucose monitoring, and pharmacologic interventions such as insulin therapy, presents an additional psychological stressor [52]. Furthermore, the heightened risk of obstetric complications, including preterm birth, preeclampsia, macrosomia, and neonatal hypoglycemia, exacerbates maternal anxiety. The necessity for frequent medical surveillance, encompassing ultrasonography and laboratory assessments, further amplifies stress levels.

Macrosomia, a direct fetal consequence of GDM, occurs due to the excessive transfer of maternal glucose across the placenta [4,53,54,55,56,57]. In response, the fetal pancreas undergoes compensatory hyperplasia, resulting in hyperinsulinemia [58]. This state promotes accelerated fetal growth by enhancing glucose uptake and lipid storage, increasing the risk of birth trauma, neonatal hypoglycemia, and long-term metabolic disturbances. The phenomenon of fetal programming suggests that exposure to hyperglycemia in utero predisposes offspring to obesity, insulin resistance, and type 2 diabetes (T2D) in later life, perpetuating an intergenerational cycle of metabolic disease [59,60,61].

Epidemiological studies, such as the Hyperglycemia and Adverse Pregnancy Outcome (HAPO) study, have contributed significantly to our understanding of the substantial links between GDM and adverse health outcomes for both the mother and child [62]. In children, GDM increases the long-term risk for the development of obesity, cardiovascular disease, and T2D [6]. A noteworthy maternal risk is the heightened likelihood of future development of diabetes. Approximately 50% of women with GDM will develop T2D within 10 to 20 years post-pregnancy and around 5.7% will develop type 1 diabetes (T1D) between 1 and 5 years post-pregnancy [63]. It is imperative to note that the progression of GDM to T1D involves a distinct mechanism of action when compared to GDM which progresses to T2D. Specifically, GDM evolving into T1D is characterized by autoimmunity and rapid *β*-cell destruction, resulting in an abrupt onset of hyperglycemia [64,65,66,67,68].

The objective of this review article is to provide an overview on our current understanding of GDM, specifically its pathophysiology, prevalence, and hormonal and metabolic changes that contribute to its development. By synthesizing the existing literature, this review aims to discuss gaps in knowledge and suggests future research directions, ultimately contributing to improved care and outcomes for women with GDM and their offspring.

## 2. Search Strategy

The search strategy for this narrative review was designed utilizing the key terms such as “gestational diabetes mellitus”, “GDM”, “glucose intolerance”, “maternal health”, “neonatal outcomes”, “hyperglycemia”, “insulin resistance”, “dietary intervention”, “placental hormones”, “inflammatory markers in pregnancy”, “genetic predisposition”, “screening methods for GDM”, and “beta-cell dysfunction”. We searched multiple databases, including PubMed, Scopus, and Web of Science, to gather a wide range of peer-reviewed articles on the topic. Only studies published in English were included to maintain consistency and accessibility of content. Additionally, reference lists of selected articles were reviewed to identify any relevant studies not captured in the initial search.

The selection strategy focused on studies addressing GDM’s pathophysiology, screening methods, diagnostic criteria, and management strategies. Given the broad scope, our goal was to provide a balanced overview to cover critical aspects of GDM’s impact on maternal and fetal health, as well as emerging trends in clinical management.

## 3. Mechanistic Insights into the Progression from GDM to T1D and T2D

Gestational diabetes mellitus (GDM) represents one of the most significant risk factors for the future development of T2D [1,69,70,71,72]. This association highlights the critical need for heightened vigilance in detecting and managing hyperglycemia during pregnancy, as such efforts could provide an opportunity for early intervention and potentially alter the trajectory of long-term metabolic complications. Pregnancy itself is a state of heightened insulin resistance, driven by placental hormones such as human placental lactogen and progesterone, which are necessary to ensure sufficient glucose supply to the growing fetus. For most women, the compensatory mechanisms of increased insulin secretion from pancreatic β-cells are sufficient to maintain euglycemia. However, in those with underlying β-cell dysfunction, this increased demand surpasses the β-cell’s compensatory capacity, leading to GDM. Therefore, GDM serves not only as a marker of transient hyperglycemia during pregnancy but also as an early clinical indicator of ongoing β-cell dysfunction, which places these women at a significantly higher risk for T2D later in life [73,74].

Understanding the mechanisms driving this progression from GDM to T2D is critical, as it may provide valuable insights for developing targeted interventions to slow or halt the global T2D epidemic. It has been hypothesized that most women who develop GDM have an inherent chronic β-cell defect that is often subclinical and becomes overt only when challenged by the metabolic demands of pregnancy. An inherent chronic β-cell defect refers to an underlying and often subclinical impairment in pancreatic β-cell function that predisposes individuals to glucose intolerance, which becomes clinically evident during periods of increased metabolic demand, such as pregnancy [75]. In most healthy pregnancies, pancreatic β-cells adapt by increasing insulin secretion to compensate for pregnancy-induced insulin resistance. However, in women with an inherent β-cell defect, this compensatory mechanism is insufficient due to reduced β-cell mass, impaired glucose-stimulated insulin secretion, and dysfunctional insulin signaling [76]. This defect is largely driven by genetic predisposition, with polymorphisms in genes such as *HNF1A*, *HNF4A*, *TCF7L2*, and *MTNR1B*, which regulate β-cell proliferation and insulin transcription, being strongly associated with both GDM and progression to T2D. Additionally, epigenetic modifications caused by early-life environmental exposures, such as maternal hyperglycemia or fetal malnutrition, can lead to persistent β-cell dysfunction [77]. Mitochondrial dysfunction, oxidative stress, and chronic low-grade inflammation further exacerbate β-cell failure by inducing apoptosis, impairing ATP production and insulin exocytosis [78,79,80]. Another critical factor is an impaired incretin response, characterized by decreased glucagon-like peptide-1 (GLP-1) and glucose-dependent insulinotropic polypeptide (GIP) activity, which results in suboptimal postprandial insulin release [81]. During pregnancy, as insulin resistance intensifies due to placental hormones (human placental lactogen, estrogen, and progesterone), the inability of β-cells to sufficiently augment insulin secretion leads to GDM [82]. Postpartum, insulin sensitivity typically improves, leading to the resolution of hyperglycemia in most cases. However, as β-cell dysfunction persists, women with GDM remain at a significantly higher risk of developing T2D. This highlights the importance of identifying and addressing β-cell dysfunction early through targeted interventions, including incretin-based therapies, β-cell preservation strategies, and metabolic lifestyle modifications, to prevent long-term metabolic deterioration.

However, it is still not clear why some women experience GDM in one pregnancy but not in a subsequent pregnancy. Several factors may contribute to the absence of GDM in later pregnancies. Improvements in lifestyle, such as adopting a healthier diet and increasing physical activity, can enhance insulin sensitivity and glucose metabolism, reducing the likelihood of GDM recurrence [83,84,85,86]. Additionally, hormonal fluctuations in a subsequent pregnancy may result in lower insulin resistance, thereby mitigating the risk [87]. Variations in placental size and function can also play a role, as the placenta influences maternal glucose metabolism through hormone secretion [88,89,90]. Differences in diagnostic thresholds, testing methodologies, or the timing of glucose tolerance testing between pregnancies could further contribute to the observed discrepancy. Lastly, the use of medications such as metformin or myo-inositol, known to improve insulin sensitivity and glucose regulation, might prevent the recurrence of GDM [91,92,93,94]. These factors highlight the complex interplay of metabolic, hormonal, and clinical influences in determining the presence or absence of GDM across pregnancies.

Moreover, recent evidence suggests that the predisposition to GDM and subsequent T2D may have developmental origins [35]. Developmental abnormalities in β-cells refer to structural and functional impairments that hinder their ability to adapt to metabolic demands, particularly during pregnancy. One key aspect of these abnormalities is a reduced β-cell mass, which may result from impaired β-cell proliferation, increased apoptosis, or a failure of β-cell neogenesis. This can be attributed to genetic factors, such as mutations in *HNF1A*, *HNF4A*, and *PDX1*, which regulate β-cell development and insulin transcription as discussed previously. Additionally, epigenetic modifications caused by intrauterine exposure to hyperglycemia, maternal malnutrition, or environmental stressors can lead to long-term β-cell dysfunction by altering gene expression involved in β-cell growth and survival. Another contributor is chronic low-grade inflammation, where elevated levels of pro-inflammatory cytokines, including TNF-α, IL-6, and IL-1β, disrupt β-cell function by impairing insulin secretion and promoting oxidative stress. This inflammatory milieu can also interfere with insulin signaling pathways, leading to defective GLUT4 translocation and reduced glucose uptake in peripheral tissues. Moreover, abnormalities in cell membrane receptors, such as insulin receptors and incretin receptors (GLP-1R, GIPR), further exacerbate β-cell dysfunction by limiting their responsiveness to insulinotropic signals. Mitochondrial dysfunction, characterized by defective ATP production, also plays a crucial role in reducing glucose-stimulated insulin secretion. Collectively, these factors contribute to an inadequate β-cell compensatory response during pregnancy, leading to GDM, and increasing the long-term risk of β-cell failure and progression to T2D.

While the relationship between GDM and T2D is well-documented, the potential link between GDM and T1D is an emerging area of research that warrants further exploration. Pregnancy induces a state of immune tolerance to prevent maternal rejection of the semi-allogeneic fetus; however, certain mechanisms may still contribute to the production of β-cell autoantibodies. One possibility is that pregnancy-related hormones, such as human placental lactogen (hPL), estrogen, and progesterone, increase insulin demand, leading to heightened β-cell activity and metabolic stress [95,96]. Estrogen and progesterone exert distinct effects on insulin secretion and sensitivity, influencing the adaptive mechanisms of β-cells during pregnancy [97]. Estrogen enhances insulin sensitivity by improving glucose uptake in peripheral tissues and stimulating β-cell proliferation, thereby facilitating better glycemic control [98,99]. In contrast, progesterone promotes insulin resistance, possibly through interference with insulin receptor signaling and impaired GLUT4 translocation, necessitating greater insulin secretion from β-cells to maintain glucose homeostasis [100]. The combined effects of these hormones create a dynamic metabolic environment that places considerable stress on pancreatic β-cells. In individuals with underlying β-cell vulnerabilities, this heightened metabolic demand may exacerbate β-cell dysfunction, leading to apoptosis or necrosis. The subsequent release of intracellular antigens into circulation can activate autoreactive T cells, triggering an immune response that promotes the production of autoantibodies against β-cells. Furthermore, pregnancy-associated immune shifts, including alterations in regulatory T cell function and the postpartum immune rebound, may facilitate a loss of self-tolerance, increasing the risk of autoimmune β-cell destruction. This interplay of metabolic and immune factors suggests that pregnancy could serve as a physiological stressor that unmasks an underlying autoimmune predisposition, potentially accelerating the development of T1D in susceptible individuals. Further research is necessary to elucidate the molecular pathways linking GDM to T1D and to identify predictive biomarkers that could aid in early detection and intervention strategies for autoimmune diabetes in women with a history of GDM.

A subset of women with GDM appear to follow a trajectory toward T1D rather than T2D, suggesting an autoimmune component underlying their β-cell dysfunction [101,102]. This autoimmune destruction of β-cells mimics processes observed in childhood-onset T1D, with pregnancy potentially serving as a trigger that unmasks or accelerates the autoimmune response in genetically predisposed individuals. The immunological landscape of GDM is characterized by dysregulated immune cell populations, including altered natural killer (NK) cell activity and shifts in T-cell subsets, which contribute to the inflammatory environment observed in affected individuals [103,104,105,106,107,108]. Studies indicate that GDM is linked to an increased proportion of cytotoxic CD27^−^CD11b^+^ NK cells and a decrease in the less mature CD27^−^CD11b^−^ NK cell subset, both in the decidua and peripheral blood [103]. These changes suggest that hyperglycemia may influence NK cell distribution and cytotoxic activity, potentially exacerbating placental dysfunction and immune dysregulation. Additionally, women with GDM exhibit increased proportions of T-helper 2 (Th2) and T-helper 17 (Th17) cells, indicative of a shift toward a more inflammatory immune state that persists postpartum [109]. The presence of T1D-associated autoantibodies in some women with GDM further supports an underlying autoimmune mechanism, with longitudinal studies revealing a 2.65-fold increased risk of impaired glucose regulation in those exhibiting these markers [67]. This finding highlights the persistence of β-cell autoimmunity beyond pregnancy and highlights the need for early identification of at-risk individuals. The heightened pro-inflammatory environment in GDM, coupled with an underlying susceptibility to autoimmunity, may not only drive postpartum β-cell dysfunction but also contribute to long-term metabolic consequences. Understanding these immune alterations in the context of GDM progression is essential for developing targeted interventions aimed at preserving β-cell function and mitigating the risk of future T1D development.

The presence of T1D-associated autoantibodies in women with GDM has profound implications for clinical care and long-term risk stratification. Studies estimate that the likelihood of developing T1D within two years postpartum varies based on the number of autoantibodies detected: approximately 17% with one autoantibody, 61% with two, and a staggering 84% with three autoantibodies [110]. Among the various autoantibodies, glutamic acid decarboxylase autoantibodies (GADA) have been shown to exhibit the highest sensitivity (63%) for predicting future autoimmune diabetes [65]. However, while GADA is valuable as a biomarker, its predictive power is limited, necessitating further research to identify additional biomarkers or combinations thereof that could enhance risk prediction.

## 4. Genetic Risk Factors for GDM

Genetics has been found to play an important role in the predisposition of GDM development [111]. A collection of genetic risk variants can be compiled into a polygenic risk score (PRS), which offers an estimation of the genetic risk impact from across the entire genome [112]. PRSs are calculated as a weighted sum of risk alleles an individual carries, where each allele’s weight is determined by its effect size identified in genome-wide association studies (GWAS) [113,114]. PRSs have been particularly useful in predicting diseases such as GDM and T2D, often using genetic variants associated with T2D due to their shared pathophysiology [115]. While PRSs can enhance disease risk stratification, their discriminative power has been modest but improves when combined with clinical parameters.

Single-nucleotide polymorphisms (SNPs) have been linked in several studies to an increased risk for GDM development in different populations worldwide (Table 1) [116]. For example, in the Western Pacific and South America, the C allele from the SNP rs780094 on the glucokinase regulator (*GCKR*) gene has been observed more frequently in GDM patients. This gene plays a key role in glycogen and triglyceride synthesis, and its dysregulation contributes to metabolic dysfunction [111]. Similarly, in South Korea and Middle Eastern GDM patients, the rs2237895 SNP in the potassium voltage-gated KCNQ1 gene is more prevalent, likely affecting β-cell function by reducing calcium efflux and impairing insulin secretion [111]. Additionally, variations in the melatonin receptor 1B (*MTNR1B*) gene, including the T allele SNP rs1387153 and G allele SNP rs10830963, have been identified in European, North American, and Western Pacific populations. These SNPs influence insulin secretion, glucose metabolism, and circadian rhythm regulation, all of which are implicated in GDM pathogenesis [111].

Furthermore, variations in genes encoding incretin receptors, insulin receptors, adiponectin, and cytokines have also been studied for their association with GDM risk. SNPs in *TCF7L2*, such as rs7903146, have been consistently linked to GDM, given the gene’s central role in the Wnt signaling pathway and β-cell function [117,118,119]. Similarly, rs7756992 in *CDKAL1*, a gene regulating β-cell development, has been associated with reduced insulin secretion in GDM patients [120,121,122,123]. Polymorphisms in *IGF2BP2*, such as rs4402960, have been linked to impaired insulin-like growth factor signaling, further predisposing individuals to GDM [124,125]. Additionally, SNPs in *SUCNR1*, a gene encoding succinate receptor 1, have been implicated in placental metabolism and insulin resistance, with increased succinate levels observed in GDM patients [126,127,128].

A study in Saudi Arabia also found an association between SNPs in the *ADIPOQ* gene, which encodes adiponectin, a key regulator of insulin sensitivity and lipid metabolism. Specifically, rs1501299 and rs2241766 have been associated with both GDM and T2D, suggesting that altered adipokine signaling contributes to insulin resistance during pregnancy [129,130]. Additionally, SNPs in genes encoding cytokines such as TNF-α (rs1800629) and IL-6 (rs1800795) have been implicated in the chronic low-grade inflammation characteristic of GDM, exacerbating insulin resistance and metabolic dysfunction [131,132]. SNPs in genes encoding incretin receptors, such as GLP1R (rs6458093), have also been linked to impaired GLP-1 signaling, which is essential for glucose-dependent insulin secretion [133].

In addition to SNPs, it is estimated that around 5% of GDM cases may be due to Maturity-Onset Diabetes in the Young (MODY). This is caused by a monogenic mutation which disrupts the physiologic response to changes in glucose levels [134]. The four most common mutations found in GDM patients occur in *HNF1A*, *HNF4A*, *GCK*, and *HNF1B* genes and affect the hepatocyte nuclear factors and glucokinase [119,135]. Hepatocyte nuclear factors (HNFs) are a family of transcription factors that play a crucial role in regulating gene expression involved in glucose metabolism, pancreatic β-cell function, and insulin secretion [136,137]. Among them, *HNF1A*, *HNF4A*, and *HNF1B* are particularly significant in the context of β-cell development and insulin regulation [138,139]. *HNF1A* and *HNF4A* are essential for the maintenance of β-cell mass and insulin gene transcription, ensuring adequate insulin production in response to glucose stimulation [140,141]. Mutations or polymorphisms in these genes have been linked to impaired β-cell function, contributing to dysregulated insulin secretion and glucose intolerance, which are hallmarks of GDM. Variants in *HNF* have been associated with an increased risk of GDM, as they impair insulin synthesis and β-cell compensatory response [142,143,144,145]. Defects in *HNF*s may result in reduced insulin secretion, altered glucose sensing, and increased hepatic glucose production, thereby exacerbating insulin resistance commonly observed during pregnancy [146].

Autoimmune GDM risk ranges from 2% to 17% and is dependent on genetics and ethnicity [65]. Vulnerability to disease is associated with the number of genes that encode for autoantibodies. Specifically, there is a significant correlation between the presence of autoantibodies after childbirth and the development of latent autoimmune diabetes in adults (LADA) [65]. Studies indicate elevated GDM-to-T2D risk in East Asian women, while Finnish and Sardinian women show higher autoimmune GDM prevalence [102,147,148]. The five key genes associated with GDM in general are transcription factor 7-like 2 (*TCF7L2*) [149,150]; melatonin receptor 1B (*MTNR1B*) [151,152]; CDK5 regulatory subunit-associated protein 1-like 1 (*CDKAL1*) [122,153]; potassium voltage-gated channel, KQT-like subfamily, member 1 (*KCNQ1*) [154,155]; and insulin receptor substrate-1 (*IRS1*) [147,156,157]. Additionally, misfunctions in the insulin-like growth factor 2 mRNA-binding protein 2 (*IGF2BP2*) have been significantly associated with the risk of decreased insulin secretion [158,159] (Table 2). These genetic variations impact genotypic and phenotypic inheritance, influencing GDM risk factors like obesity and glucose intolerance [147]. These genetic markers not only enhance our understanding of the pathophysiological mechanisms underlying GDM but also demonstrate potential for application in clinical settings.

A promising approach involves the development of Genetic Risk Scores (GRS), which aggregate the effects of multiple genetic variants to quantify an individual’s genetic predisposition to GDM. Studies have demonstrated that incorporating GRS derived from GDM-associated genes can improve the predictive accuracy of traditional risk factors such as maternal age, BMI, and family history [161,162,163,164,165,166]. By integrating GRS into predictive models, healthcare providers could identify high-risk individuals earlier in pregnancy or even pre-conception, enabling timely lifestyle interventions and closer clinical monitoring. The utility of GRS extends to its potential role in population-wide screening programs. Genetic markers associated with GDM could serve as a non-invasive screening tool to stratify patients based on their risk profiles. This stratification would allow targeted interventions, optimizing healthcare resources while reducing the burden of adverse outcomes. Importantly, GRS could also complement biomarkers and clinical parameters, creating a robust predictive framework for comprehensive GDM risk assessment.

To translate these genetic insights into routine clinical practice, future research must validate the predictive performance of GRS across diverse populations, accounting for genetic heterogeneity and environmental interactions. Additionally, studies should explore the cost-effectiveness and ethical considerations of incorporating genetic screening into standard prenatal care. As the field advances, GRS and other genetic tools hold promise for transforming GDM management, paving the way for personalized prevention strategies and improved maternal and fetal outcomes.

## 5. Pathophysiology of GDM and Association with T1D and T2D

### 5.1. Molecular Mechanisms Underlying GDM

**Figure 2:**
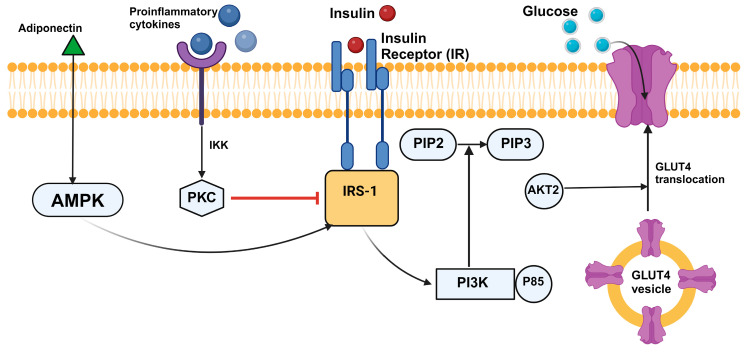
A schematic representation of insulin signaling: When insulin binds to its receptor (IR), it triggers activation of IRS-1. Adiponectin enhances activation of IRS-1 via the AMP-activated protein kinase (AMPK) pathway. Activated IRS-1 then stimulates phosphatidylinositol-3-kinase (PI3K), which converts phosphatidylinositol-4, 5-bisphosphate (PIP2) into phosphatidylinositol-3-, 4-, 5-phosphate (PIP3). PIP3 subsequently activates Akt2, leading to the translocation of GLUT4 transporters to the cell surface and facilitating glucose entry into the cell. On the other hand, pro-inflammatory cytokines activate protein kinase C (PKC) through the IκB kinase (IKK), which then inhibits IRS-1 leading to disruption in insulin signaling. Created in BioRender. https://BioRender.com/a92r994 (accessed on 2 December 2024).

Insulin resistance is one of the key mechanisms that has been implicated in the pathophysiology of GDM [160,167,168,169,170]. Insulin resistance is a critical health issue in which the cells become less responsive to insulin [155,156,157,158,159,160,167,168,169,170,171,172,173,174]. One of the crucial players in the development of insulin resistance is glucose transporter type 4 (GLUT4), which is intricately involved in the body’s ability to manage glucose. GLUT4 is a glucose transporter found mainly in adipose tissue and muscle, which are vital for energy storage and use [175,176,177]. In normal physiology, insulin binds to its receptor on the cell surface, triggering a series of intracellular cascades that lead to the translocation of GLUT4 from intracellular vesicles to the cell membrane (Figure 2) [178]. Once at the membrane, GLUT4 facilitates the entry of glucose into the cell, thereby lowering blood glucose levels. However, in conditions of insulin resistance, the efficiency of this process is significantly compromised. The signaling pathway that normally results in the movement of GLUT4 to the cell membrane is impaired. Despite the presence of insulin, the cells do not respond adequately, leading to diminished glucose uptake. As a result, glucose remains in the bloodstream, leading to hyperglycemia. Over time, this can place increased demand on the pancreas to produce more insulin, potentially leading to β-cell exhaustion and the onset of diabetes. The dysfunction of GLUT4 not only impedes glucose management in the body but also reflects the complex interplay between various metabolic pathways affected by insulin resistance. Factors such as pro-inflammatory cytokines can interfere with the insulin signaling pathways, further exacerbating the impaired translocation and function of GLUT4 (Figure 2).

In a healthy pregnancy, many metabolic changes take place to ensure a hormonal and electrolyte balance for both mother and child. These changes include an increase in the basal endogenous glucose production by 30% to meet fasting energy needs [35]. This heightened production is driven by an increased maternal physiological demand, the presence of placental hormones, and an increase in hormones. In addition to the elevated glucose levels, insulin sensitivity decreases by approximately 50%, but is accompanied by a two- to three-fold rise in insulin secretion from the β cells [170]. The relatively rapid rise in maternal caloric intake and weight over the nine-month period necessitates elevated insulin requirements to adequately perfuse the newly developing tissues [179]. Consequently, even healthy pregnancies may be associated with a small degree of insulin resistance during the second and third trimesters [180].

Placental hormones such as human placental lactogen (hPL) and human placental growth hormone (hPGH) have been identified as contributors to the increased insulin resistance [181,182,183]. This is important as it maintains a glucose gradient between the mother and the fetus [184]. Additionally, the increased estrogen, progesterone, and cortisol produced by the mother exacerbate this gradient [181]. As pregnancy advances, the fetus will divert increasing amounts of glucose towards itself, further necessitating insulin resistance and hepatic glucose production in the mother [67].

**Figure 3:**
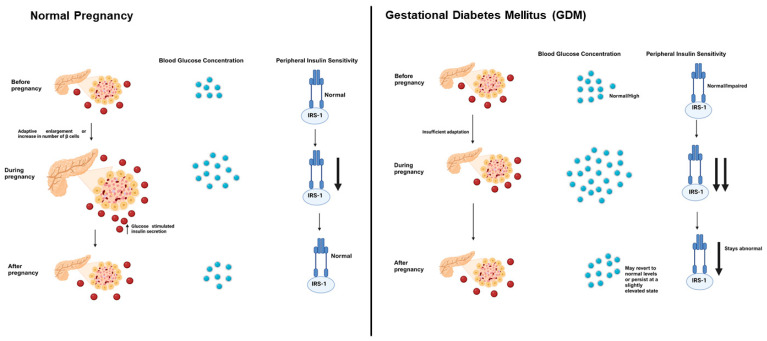
A comparison of β-cell function and insulin sensitivity in normal and GDM pregnancies: During normal pregnancy, β-cells undergo expansion through hyperplasia and hypertrophy to accommodate the increased metabolic requirements. Concurrently, there is an increase in blood glucose levels due to reduced insulin sensitivity. After pregnancy, β-cells, blood glucose, and insulin sensitivity generally revert to their original state. However, in GDM, β-cells are unable to adequately adapt to the demands of pregnancy, causing an increase in blood glucose levels due to decreased insulin sensitivity. GDM resolves postpartum as the primary drivers of insulin resistance—placental hormones such as human placental lactogen (hPL) and progesterone—are no longer present. However, residual β-cell dysfunction, persistent low-grade inflammation, and metabolic abnormalities remain in many women, increasing their long-term risk for type 2 diabetes (T2D). Created in BioRender. https://BioRender.com/o66i148 (accessed on 2 December 2024).

Pancreatic β-cells are responsible for adapting to increased insulin demand during pregnancy by enhancing insulin synthesis and secretion [35] (Figure 3). However, in GDM, β-cells fail to compensate adequately, leading to maternal hyperglycemia [185]. This β-cell dysfunction arises from a combination of hormonal, cytokine-mediated, metabolic, and genetic factors that impair insulin production and secretion [186,187,188]. Hormonal changes during pregnancy, particularly elevated levels of human placental lactogen (hPL), progesterone, cortisol, and prolactin, contribute to insulin resistance [189]. Normally, β-cells increase their function to overcome this resistance, but in GDM, an inherent β-cell defect (as discussed in previous section) or metabolic stress prevents adequate compensation [190]. Cytokines such as TNF-α, IL-6, and C-reactive protein (CRP) create a pro-inflammatory environment that directly interferes with insulin signaling and β-cell survival [191,192,193,194]. Chronic inflammation induces endoplasmic reticulum (ER) stress and oxidative stress, leading to increased β-cell apoptosis and reduced insulin gene expression [195]. Additionally, mitochondrial dysfunction reduces ATP production, impairing glucose-stimulated insulin secretion. Glucose toxicity and lipotoxicity further exacerbate β-cell dysfunction [196]. In GDM, prolonged hyperglycemia and elevated free fatty acids (FFAs) overload β-cells, impairing their function through glucolipotoxicity. Excess FFAs activate stress pathways such as protein kinase C (PKC) and JNK signaling, leading to β-cell inflammation and apoptosis [197,198,199,200].

Despite these metabolic disturbances, GDM often resolves after parturition, primarily as the hormonal and inflammatory factors that drive insulin resistance decline postpartum [185]. Placental hormones, including hPL, estrogen, and progesterone, which antagonize insulin action, are rapidly cleared from circulation after delivery, restoring insulin sensitivity [201]. Additionally, inflammatory cytokine levels, which contribute to β-cell stress and systemic insulin resistance, decrease significantly after childbirth. As insulin resistance diminishes, maternal glucose homeostasis improves, and normoglycemia is restored.

However, in women with underlying β-cell defects, chronic low-grade inflammation, or persistent insulin resistance, the resolution of GDM may be transient, with an increased risk of progression to T2D [191]. Studies indicate that up to 50% of women with a history of GDM develop T2D within 10–20 years postpartum, emphasizing that β-cell dysfunction may not fully recover even after pregnancy [185]. Identifying women at high risk through metabolic screening and genetic profiling could help implement preventive strategies, such as lifestyle modifications, incretin-based therapies, and insulin-sensitizing agents, to preserve β-cell function and prevent long-term metabolic complications.

The differences between normal pregnancy and GDM highlight the delicate balance required for maintaining glucose homeostasis [202]. In normal pregnancy, the body’s adaptive mechanisms are typically efficient in managing the metabolic demands posed by insulin resistance; for instance, refs. [203,204,205]. However, during pregnancy, the metabolic environment becomes uniquely demanding, driven by the dual needs of the mother and the developing fetus. The maternal body undergoes substantial hormonal and metabolic shifts to prioritize nutrient delivery to the fetus, a process regulated by placental hormones such as human placental lactogen, progesterone, cortisol, and prolactin [176]. These hormones play a critical role in modulating maternal metabolism, but they also induce a state of progressive insulin resistance as pregnancy advances.

In most women, the body’s compensatory mechanisms, primarily the beta-cell expansion and increased insulin secretion, are sufficient to overcome this insulin resistance and maintain glucose homeostasis [206]. However, in GDM, these mechanisms are overwhelmed or insufficient, leading to hyperglycemia. Several factors contribute to this breakdown. A key factor is pre-existing beta-cell dysfunction, which can result from genetic predisposition, age-related decline in beta-cell capacity, or prior metabolic disorders such as prediabetes or polycystic ovary syndrome (PCOS) [28]. PCOS is increasingly recognized as a condition associated with pre-existing beta-cell dysfunction, contributing to the risk of GDM [207,208]. PCOS is a complex endocrine disorder characterized by hormonal imbalances, insulin resistance, and metabolic dysfunction, with androgens, incretins, and glucagon-like peptide-1 receptor (GLP-1R) signaling playing significant roles in its pathophysiology [209]. Hyperandrogenism, a hallmark of PCOS, results from increased ovarian and adrenal androgen production, primarily driven by elevated luteinizing hormone (LH) secretion and insulin resistance [210]. Excess androgens, particularly testosterone and dihydrotestosterone (DHT), disrupt follicular development, leading to ovulatory dysfunction, anovulation, and polycystic ovarian morphology [209]. Furthermore, androgens impair GLP-1 secretion and incretin hormone activity, exacerbating insulin resistance and metabolic disturbances in PCOS [209].

Incretins, including GLP-1 and glucose-dependent insulinotropic polypeptide (GIP), are gut-derived hormones that enhance insulin secretion in response to nutrient intake [211,212,213,214]. Studies indicate that women with PCOS exhibit impaired incretin response, particularly reduced GLP-1 secretion and diminished GLP-1 receptor (GLP-1R) expression in pancreatic β-cells, contributing to insufficient postprandial insulin release and hyperglycemia [215,216,217,218,219]. GLP-1 also exerts direct anti-androgenic effects by modulating ovarian steroidogenesis and inhibiting excessive androgen production from theca cells [220,221]. Therefore, GLP-1R agonists, such as liraglutide and dulaglutide, have shown therapeutic potential in PCOS by reducing hyperandrogenemia, improving insulin sensitivity, and promoting weight loss [222,223,224,225,226,227,228,229].

Additionally, impaired incretin function in PCOS leads to glucose intolerance, compensatory hyperinsulinemia, and further androgen excess, creating a vicious cycle that perpetuates insulin resistance and reproductive dysfunction. Dysregulated incretin signaling also affects hepatic gluconeogenesis and lipid metabolism, contributing to the increased prevalence of non-alcoholic fatty liver disease (NAFLD) and metabolic syndrome in PCOS patients [230,231,232].

While insulin resistance is a hallmark of PCOS, it is important to consider whether women with PCOS but without insulin resistance are also at risk for GDM. Some studies suggest that even in the absence of insulin resistance, PCOS-related hormonal imbalances, including hyperandrogenism, chronic low-grade inflammation, and altered adipokine secretion, may contribute to beta-cell dysfunction and impaired glucose tolerance during pregnancy [233,234]. However, the data on GDM risk in insulin-sensitive PCOS patients remains limited, warranting further investigation.

Another aspect of PCOS that may influence glucose metabolism is functional hyperprolactinemia—elevated prolactin levels without an underlying prolactinoma. Increased prolactin can exacerbate PCOS symptoms such as menstrual irregularities and infertility and may also impact glucose homeostasis [235,236]. Some clinicians use low-dose dopamine agonists, such as bromocriptine or cabergoline, to lower prolactin levels, which in turn may have beneficial effects on metabolic function [237]. Dopamine agonists not only normalize prolactin levels but also improve insulin sensitivity, potentially through central mechanisms involving hypothalamic regulation of energy balance and glucose metabolism [238]. Additionally, by reducing prolactin levels, dopamine agonists may mitigate prolactin-induced hepatic glucose production and adipose tissue dysfunction, both of which are implicated in metabolic dysregulation in PCOS [239].

Regarding the potential impact of dopamine agonist therapy on GDM risk, it has been hypothesized that women with PCOS who receive dopamine agonists may have a reduced likelihood of developing GDM. This is likely due to improved insulin sensitivity and better glycemic control achieved with these medications. However, large-scale clinical trials specifically evaluating dopamine agonists in PCOS patients in relation to GDM risk are still lacking. Further research is needed to clarify the role of dopamine agonists in modulating glucose metabolism in pregnancy and to determine whether they could be a viable strategy for reducing GDM incidence in women with PCOS.

Additionally, obesity is a major contributing factor, as excess adipose tissue leads to chronic low-grade inflammation, elevated free fatty acid levels, and increased production of adipokines, all of which exacerbate insulin resistance [240]. Obesity also amplifies the pro-inflammatory environment of pregnancy, compounding the stress on insulin signaling pathways. Another important contributor is placental function itself; variations in placental size, hormone secretion, and inflammatory signaling can influence the degree of insulin resistance experienced by the mother. In some cases, factors such as pre-existing vascular dysfunction, associated with conditions like hypertension, can further impair glucose metabolism by limiting the effective delivery of nutrients and oxygen to the pancreas and other insulin-sensitive tissues.

Understanding the factors responsible for these trends is crucial, as they offer insight into why some women develop GDM while others do not, despite being exposed to similar hormonal changes. Genetic studies have identified polymorphisms associated with insulin signaling pathways and beta-cell function that may predispose certain individuals to GDM. Additionally, environmental and lifestyle factors, such as diet, physical activity, and stress, play a significant role in influencing the balance between insulin sensitivity and resistance. Exploring these intricate interactions can provide a more comprehensive understanding of the pathophysiology of GDM and inform targeted interventions to prevent or manage the condition.

### 5.2. Immunological Aspects Connecting GDM and T1D Progression

In the subgroup of the 5–10% of women who develop T1D post-pregnancy, the dysglycemia is frequently linked to the presence of autoantibodies [63]. Notably, insulin autoantibodies (IAA), tyrosine phosphatase-like islet antigen autoantibodies (IA2-A), zinc transporter 8 autoantibodies (ZnT8), and anti-glutamic acid decarboxylase antibodies (GADA) are most commonly identified in this context. The frequencies of these markers of autoimmunity vary depending on the specific autoantibody studied, the detection method employed, and the population observed [65]. Among the antibodies tested, GADA is the most widely reported with a frequency between 0 and 10% [6]. Past studies on GADA and IA2-A in GDM patients have yielded different results, with some studies identifying a higher frequency of these autoantibodies in GDM and others showing no difference between the GDM patients and controls [101,241,242,243]. Currently, however, the combination of GADA and IA2-A has an 85% sensitivity for the diagnosis of autoimmune diabetes [244]. Generally, titers for all autoantibodies are lower in patients with GDM compared to cases of newly diagnosed T1D [65]. This is because during pregnancy the immune system shifts towards a TH2-mediated immunomodulation which suppresses autoimmunity. After pregnancy, however, the TH2 cytokines decrease, causing a worsening of TH1-mediated autoimmune disorders like T1D. This means that pregnancy has been shown to be protective against autoimmunity while post-pregnancy can increase a patient’s susceptibility to autoimmunity, even decades later [245]. Consequently, autoantibody titers are more indicative of a slow-developing autoimmune process similar to those with LADA [246].

### 5.3. Long-Term Effects of Hyperglycemia During Pregnancy on Pancreatic Function

At the beginning of pregnancy, a low fetal blood glucose level allows maternal blood glucose to remain at a normal level and still maintain a gradient [247]. However, as pregnancy progresses, maternal glucose must increase to uphold this same gradient as the fetus uses more glucose to grow rapidly [247]. Conversely, to protect the fetus from excessively high glucose levels, such as following a meal, maternal pancreatic β-cells must develop an increased capacity to respond to acute increases in maternal blood glucose [248]. Any dysfunction in this regulatory mechanism can have negative long-term side effects for pancreatic function in both the fetus and the mother.

GDM is closely linked with metabolic changes including hyperglycemia, inflammation, hyperinsulinemia, hyperleptinemia, and dyslipidemia [249]. These factors affect a mother’s adaptation to pregnancy as well as the health of the fetus [63]. In addition to increased incidence of diabetes and obesity later in life, women with a history of GDM have a seven-fold increased risk of pancreatic cancer [250,251].

The heightened generation of reactive oxygen species in hyperglycemia is believed to damage the pancreatic cell DNA and overwhelm the antioxidant mechanisms. This results in a loss of function in crucial proteins for pancreatic function [251]. Prolonged exposure to elevated glucose levels affects the developing fetus, causing increased stimulation of fetal β-cells. This leads to hyperinsulinemia in the fetus, which has been shown to impair neuronal and cardiac development as well as lead to pancreatic exhaustion early in life [250].

While the role of reactive oxygen species (ROS) in hyperglycemia-induced DNA damage is well-documented, the direct link to specific genes implicated in GDM onset, such as *IRS1*, *CDKAL1*, *MTNR1B*, and *GCK*, remains an area of active research. These genes, known to regulate glucose metabolism, insulin signaling, and β-cell function, may also be susceptible to oxidative stress-induced modifications. Exploring the connection between oxidative DNA damage and these genetic factors could reveal key molecular pathways driving GDM, offering insights into how maternal hyperglycemia impacts fetal health and β-cell integrity. Such understanding may guide the development of targeted therapies to reduce oxidative stress and mitigate adverse outcomes for both mother and child.

### 5.4. Immunological Mechanisms in GDM

Pregnancy requires a complex and tightly regulated immune response to ensure the survival of the semi-allogeneic fetus, which contains both maternal and paternal antigens [252]. This immunological balance is achieved through a profound shift from a Th1 (pro-inflammatory) to a Th2 (anti-inflammatory) cytokine profile, which reduces the likelihood of fetal rejection [253]. Regulatory T cells (Tregs) play a crucial role in maintaining this balance by suppressing excessive maternal immune responses and promoting an environment conducive to fetal tolerance [254]. However, in GDM, this equilibrium may be disturbed, resulting in a pro-inflammatory state that exacerbates insulin resistance and glucose dysregulation.

Emerging evidence indicates that women with GDM exhibit elevated levels of pro-inflammatory cytokines, including tumor necrosis factor-alpha (TNF-α), interleukin-6 (IL-6), and interleukin-1β (IL-1β) [255,256]. These cytokines, produced by activated maternal immune cells and the placenta, contribute to systemic inflammation and directly interfere with insulin signaling pathways [257]. TNF-α, for instance, has been shown to inhibit insulin receptor substrate-1 (IRS-1) phosphorylation, thereby impairing glucose uptake in peripheral tissues [258,259,260]. Similarly, IL-6 and IL-1β amplify the inflammatory cascade and further disrupt metabolic homeostasis [261]. This inflammatory milieu creates a vicious cycle, wherein heightened inflammation leads to worsening insulin resistance, which in turn exacerbates hyperglycemia and perpetuates the inflammatory state.

In addition to cytokine dysregulation, hormonal changes during pregnancy significantly influence immune function. Progesterone and estrogen, which are elevated during pregnancy, modulate immune cell activity by promoting Th2 polarization and enhancing Treg expansion [262,263,264,265,266]. These hormonal adaptations are essential for creating an immune-tolerant environment. However, in GDM, hormonal dysregulation may impair these protective mechanisms, shifting the immune response toward a pro-inflammatory state. Placental-derived hormones, such as human placental lactogen (hPL) and cortisol, may also contribute to this dysregulation by inducing insulin resistance and altering maternal immune cell activity [267,268].

The placenta itself acts as an immunological organ, producing cytokines, chemokines, and extracellular vesicles that influence maternal–fetal interactions. In GDM, placental inflammation and oxidative stress are often observed, further exacerbating systemic inflammation [269]. Placental macrophages (Hofbauer cells) and trophoblasts are key sources of these inflammatory mediators, which not only impair maternal glucose regulation but may also impact fetal development. For example, excessive placental inflammation has been associated with altered fetal growth patterns, predisposing offspring to metabolic disorders later in life.

This interplay between immune dysregulation and glucose metabolism in GDM highlights the critical need for targeted interventions. Potential therapeutic strategies include the use of anti-inflammatory agents, such as TNF-α inhibitors or IL-6 receptor blockers, to mitigate systemic inflammation and improve insulin sensitivity. Additionally, interventions aimed at enhancing Treg function, such as low-dose interleukin-2 therapy or the use of probiotics to modulate gut microbiota, hold promise in restoring immune balance. The exploration of immunomodulatory therapies tailored to the unique immune profile of GDM patients could significantly improve outcomes for both mothers and their offspring.

Furthermore, longitudinal studies are needed to elucidate the long-term consequences of immune dysregulation in GDM. The increased risk of T2D and cardiovascular disease observed in women with a history of GDM may, in part, be driven by persistent low-grade inflammation. Similarly, offspring exposed to a pro-inflammatory intrauterine environment are at an increased risk of obesity, insulin resistance, and T2D, underscoring the intergenerational impact of immune dysfunction in GDM. By advancing our understanding of the immune mechanisms underlying GDM, researchers and clinicians can develop more precise diagnostic tools and personalized therapeutic approaches to address this multifaceted condition.

### 5.5. Role of Placental Hormones and Signaling in GDM

The placenta is a dynamic organ that serves as the critical interface between the mother and fetus, orchestrating a multitude of physiological processes to support fetal growth while maintaining maternal metabolic and immunological adaptations [270]. Its ability to secrete a range of hormones, cytokines, growth factors, and other bioactive molecules ensures the proper progression of pregnancy [270,271,272]. In GDM, however, these functions may become dysregulated, contributing to both maternal and fetal complications [273,274,275].

Key placental hormones such as human placental lactogen (hPL), human placental growth hormone (hPGH), progesterone, and cortisol are essential for maintaining maternal–fetal tolerance and ensuring nutrient supply to the fetus. These hormones play a central role in inducing maternal insulin resistance, which is a normal adaptation during pregnancy designed to prioritize glucose transfer to the fetus [203]. By reducing maternal glucose uptake in peripheral tissues, these hormones maintain a glucose gradient that facilitates efficient placental transfer to the developing fetus. However, in GDM, excessive secretion of these hormones exacerbates maternal insulin resistance, placing a significant burden on pancreatic β-cells. When β-cell compensatory mechanisms fail to meet the increased insulin demand, hyperglycemia develops, characterizing the hallmark metabolic disruption of GDM.

In addition to hormonal signaling, the placenta is a source of extracellular vesicles (EVs), including exosomes, which carry proteins, lipids, and nucleic acids such as microRNAs (miRNAs) [276]. These EVs act as mediators of cell-to-cell communication and can influence maternal metabolism and immune responses [277]. Placental-derived exosomes in GDM have been shown to carry inflammatory mediators that exacerbate systemic inflammation and insulin resistance. For instance, exosomes enriched with specific miRNAs, such as miR-16 and miR-210, have been implicated in disrupting insulin signaling pathways and promoting a pro-inflammatory state [278]. These exosomes can also alter the expression of key genes in maternal tissues, further aggravating the metabolic disturbances associated with GDM.

A critical aspect of placental dysfunction in GDM is the presence of oxidative stress, often driven by hyperglycemia-induced production of ROS. Elevated levels of ROS impair placental function by damaging cellular components, including lipids, proteins, and DNA. This oxidative damage disrupts normal placental processes such as angiogenesis, nutrient transport, and hormone secretion. For instance, oxidative stress can lead to the overproduction of cortisol and other stress hormones, further amplifying maternal insulin resistance. Moreover, ROS-induced damage to the mitochondrial function in placental cells can impair energy metabolism, contributing to poor placental efficiency and fetal growth abnormalities.

The role of placental inflammation is also significant in GDM. Placental macrophages (Hofbauer cells) and trophoblasts produce cytokines such as TNF-α and IL-6, which not only exacerbate maternal systemic inflammation but may also directly influence fetal development [279,280,281]. Elevated levels of these cytokines have been linked to altered placental vascularization, potentially contributing to adverse pregnancy outcomes such as preeclampsia and intrauterine growth restriction [282]. Additionally, chronic inflammation in the placenta can impair the transfer of essential nutrients, including glucose and amino acids, impacting fetal growth and metabolic programming.

Emerging research has also highlighted the role of placental epigenetics in GDM [283]. Alterations in DNA methylation, histone modification, and non-coding RNA expression in placental tissues from women with GDM have been linked to dysregulation of genes involved in metabolic and inflammatory pathways [283,284,285,286]. These epigenetic changes may not only affect the immediate function of the placenta but also have long-term consequences for the offspring, predisposing them to obesity, insulin resistance, and T2D later in life.

The interplay between placental signaling pathways and maternal metabolic adaptations in GDM underscores the complexity of this condition. Targeting placental dysfunction holds promise as a therapeutic approach. For instance, antioxidants that reduce oxidative stress, such as N-acetylcysteine, and anti-inflammatory agents that modulate cytokine production could help mitigate placental inflammation and improve maternal and fetal outcomes. Additionally, understanding the role of placental exosomes and miRNAs in GDM pathogenesis could pave the way for novel diagnostic and therapeutic strategies, including the development of biomarkers for early detection and targeted therapies to normalize placental signaling.

### 5.6. Hypothalamic Involvement in GDM

The hypothalamus, a central hub for regulating energy homeostasis, glucose metabolism, and neuroendocrine function, is emerging as a pivotal player in the pathophysiology of GDM. This small yet highly complex brain structure integrates signals from peripheral organs and circulating hormones to maintain energy balance and ensure adequate nutrient supply to the developing fetus. During pregnancy, significant metabolic adaptations are required to meet the dual demands of maternal energy needs and fetal growth. The hypothalamus orchestrates these adaptations by responding to hormonal signals such as leptin, insulin, ghrelin, and adiponectin. However, in GDM, disruptions in hypothalamic function appear to contribute to the metabolic dysregulation characteristic of the condition.

#### 5.6.1. Hormonal Regulation and Hypothalamic Dysfunction in GDM

Leptin and insulin are key hormones that influence hypothalamic activity [287,288]. Leptin, secreted by adipose tissue, acts on the hypothalamus to suppress appetite and increase energy expenditure [289]. During pregnancy, leptin resistance is a normal physiological adaptation that allows for increased caloric intake to support fetal growth. However, in GDM, excessive maternal adiposity and chronic hyperleptinemia may lead to profound leptin resistance in the hypothalamus, exacerbating dysregulated appetite control and promoting overeating [290]. Similarly, insulin, which normally acts on hypothalamic neurons to inhibit hepatic glucose production and regulate appetite, may lose its efficacy in the context of GDM due to hypothalamic insulin resistance [291]. This resistance is partly driven by elevated levels of pro-inflammatory cytokines, such as TNF-α and IL-6, which impair insulin receptor signaling within the hypothalamus.

Ghrelin, a hormone produced by the stomach, stimulates appetite and is tightly regulated during pregnancy [292]. Altered ghrelin levels in GDM may further contribute to hypothalamic dysfunction, leading to increased food intake and weight gain. Additionally, adiponectin, an anti-inflammatory hormone secreted by adipose tissue, typically enhances hypothalamic insulin sensitivity [293,294]. Reduced adiponectin levels observed in GDM may exacerbate hypothalamic resistance to metabolic hormones, compounding the dysregulation of energy homeostasis.

#### 5.6.2. Hypothalamic Inflammation and Its Role in GDM

Chronic low-grade inflammation in the hypothalamus is a hallmark feature in metabolic disorders such as obesity and T2D and is increasingly recognized in GDM [295]. This inflammation is characterized by microglial activation and the production of pro-inflammatory cytokines, which disrupt neuronal signaling pathways involved in glucose and energy regulation [296]. Elevated circulating glucose levels in GDM may further contribute to hypothalamic inflammation through glucose toxicity. This process involves the overproduction of reactive oxygen species (ROS) and activation of stress pathways in hypothalamic neurons, leading to cellular damage and impaired function.

Animal models have demonstrated that maternal hyperglycemia can induce hypothalamic inflammation not only in the mother but also in the offspring [297]. For example, studies have shown that offspring exposed to a hyperglycemic intrauterine environment exhibit altered hypothalamic development, including impaired leptin and insulin signaling pathways. This disruption predisposes the offspring to metabolic disorders such as obesity, insulin resistance, and T2D later in life, highlighting the intergenerational impact of hypothalamic dysfunction in GDM.

#### 5.6.3. Neuroendocrine Regulation and Glucose Toxicity

The hypothalamus also regulates the hypothalamic–pituitary–adrenal (HPA) axis, which plays a critical role in stress responses and glucose metabolism. In GDM, dysregulation of the HPA axis may lead to excessive cortisol production, further contributing to insulin resistance and hyperglycemia [298]. Additionally, glucose toxicity in the hypothalamus may impair the production of neuropeptides such as pro-opiomelanocortin (POMC) and neuropeptide Y (NPY), which are crucial for regulating appetite and energy balance [299]. This impairment creates a feedback loop that perpetuates metabolic dysfunction, exacerbating the severity of GDM.

Understanding the precise mechanisms of hypothalamic involvement in GDM could open new avenues for therapeutic intervention. Anti-inflammatory strategies targeting hypothalamic inflammation, such as the use of specific cytokine inhibitors or microglial modulators, may help restore normal hypothalamic function. Neuroprotective agents, including antioxidants that reduce ROS production, could mitigate glucose toxicity and preserve neuronal integrity in the hypothalamus. Additionally, therapies aimed at improving leptin and insulin sensitivity in the hypothalamus, such as adiponectin analogs or central nervous system-penetrating insulin sensitizers, may enhance metabolic regulation.

Behavioral interventions, including dietary modifications and physical activity, also have the potential to improve hypothalamic function by reducing systemic inflammation and improving hormonal signaling. Emerging technologies, such as non-invasive brain stimulation techniques, could provide innovative approaches to modulate hypothalamic activity and improve metabolic outcomes in GDM [300].

## 6. Clinical Manifestations and Complications

### 6.1. Clinical Presentation of GDM

Typically, GDM does not exhibit noticeable symptoms early in pregnancy. This may be due to the milder form of hyperglycemia in comparison to T1D and T2D [301]. Some women, however, do experience more significant increases in glucose and present similarly to patients with T2D with polyuria, polydipsia, and polyphagia [302].

Polyphagia, excessive hunger, is a well-known phenomenon in diabetes, occurring despite hyperglycemia due to the inability of cells to effectively utilize glucose due to insulin deficiency or resistance [303]. In the context of GDM, however, increased hunger may also be influenced by elevated levels of leptin. During pregnancy, many women develop a state of leptin resistance, in which the body’s response to leptin—a hormone that normally suppresses appetite and regulates energy balance—is diminished [304]. This impaired leptin signaling can lead to increased appetite and excessive weight gain, both of which are risk factors for the development of GDM [305]. Additionally, leptin resistance in pregnancy is associated with alterations in insulin signaling, further exacerbating metabolic dysfunction [306]. While leptin is primarily produced by adipose tissue, the placenta also secretes leptin, contributing to its elevated levels during pregnancy [307]. However, in GDM, the regulatory mechanisms that normally balance energy intake and expenditure become dysregulated, reinforcing a cycle of hyperphagia, weight gain, and insulin resistance [308]. Understanding the role of leptin resistance in GDM provides insight into potential therapeutic targets, such as lifestyle modifications and pharmacological interventions aimed at improving leptin sensitivity and metabolic control during pregnancy.

Symptoms normally manifest before the 28th week of pregnancy and can be accompanied by disproportionate weight gain or change in BMI. The best way to identify GDM is through a glucose tolerance test. Currently, there is no universally accepted standard regarding screening symptoms for diagnosis of GDM; however, practitioners tend to follow the guidance of their national medical organizations. In the United States, the American Diabetes Association (ADA) recommends GDM screening for all women with risk factors or symptoms of glucose intolerance [180].

### 6.2. Potential Complications Associated with GDM

The onset of GDM heightens the risk of many complications for both the mother and the fetus. With regards to the fetus, large for gestational age (LGA) newborn and macrosomia (defined as birth weight >4000 g) as well as altered brain and eye development are some of the most common comorbidities [62]. Elevated concentrations of amino acids and fatty acids in maternal blood deliver an excess of nutrients to the fetus causing hyperinsulinemia and excessive growth of all insulin sensitive tissues [1]. Animal models also suggest that this fetal environment induces inflammation of the hypothalamus, leading to further metabolic irregularities in the offspring [309]. Consequently, the American College of Obstetricians and Gynecologists (ACOG) recommends ultrasound surveillance for all mothers with GDM throughout pregnancy [310]. Alongside increased fetal size during gestation, maternal hyperglycemia is linearly correlated with increased incidence of obesity in offspring [1]. According to the HAPO follow-up study, maternal glucose levels are inversely correlated with insulin sensitivity and *β*-cell function, contributing to a higher prevalence of glucose tolerance malfunctions [62]. This is theorized to be influenced by various factors including genetic and epigenetic inheritance and exposure to intrauterine hyperglycemia [1]. Additionally, GDM has been associated with altered retinal and brain development in the fetus. This is due to altered placental levels of the fatty acid docosahexaenoic acid transporter (NLS1), which is key for healthy development [35].

Maternal complications of GDM can be categorized as complications during pregnancy and postpartum. During pregnancy, patients face increased risk of disorders such as preeclampsia, polyhydramnios, increased risk of operative birth (cesarean or instrument-assisted vaginal), and maternal trauma from excess fetal growth [62]. These complications pose significant dangers and can potentially lead to seizures, stroke, premature birth, or death of the mother or the fetus [311]. Delivery risks are also heightened, with the HAPO study finding a direct correlation between rate of Cesarean sections and maternal glycemia, with a frequency of 23.7% [312]. Postpartum patients are at an elevated risk of developing metabolic disorders such as T1D and T2D, as well as cardiovascular disease (CVD). According to the American Heart Association (AHA), there is a two-fold higher risk of developing CVD in women with GDM [313,314]. This heightened risk is attributed to a greater likelihood of increased coronary artery calcium and carotid intima thickness, both precursors to atherosclerosis [1].

### 6.3. Gestational Influences on the Progression to DM

Pregnancy induces a reduction in insulin sensitivity, which places women with even minor impairments in pancreatic β-cell function at heightened risk for developing GDM [244]. Research has demonstrated that, even in women whose oral glucose tolerance tests normalized postpartum and remained normal for 13 months after delivery, an impaired insulin response persisted [315,316,317,318]. The severity of this impaired response was comparable to that observed in first-degree relatives of individuals with T1D who are antibody positive. These findings strongly suggest that the improvement in glucose tolerance after pregnancy is likely attributable to increased insulin sensitivity rather than a recovery in insulin production [319]. This indicates that pregnancy may unmask pre-existing β-cell dysfunction, with GDM serving as an early indicator of potential future glucose intolerance rather than directly causing diabetes. In this context, GDM may highlight underlying β-cell deficiencies that existed before pregnancy. However, the concept of partial recovery of insulin secretion following pregnancy has yet to be thoroughly evaluated [244].

## 7. Screening and Diagnosis

### 7.1. Current Screening Methods for GDM During Pregnancy

The prevalence of GDM worldwide varies significantly, in part due to the lack of standard protocols for diagnosis [320]. Early glucose screening is crucial for proper monitoring of the health of both the mother and the fetus and is normally conducted during the first prenatal visit (between 24–28 weeks) for women with risk factors [321]. Currently, there are two accepted protocol-based guidelines for diagnosis of GDM in the United States. The first test is based on guidelines from the International Association of the Diabetes and Pregnancy Study Groups (IADPSG), which uses a “one step approach”. The second test is the more traditional “two step approach” recommended by the American College of Obstetrics and Gynecology (ACOG) and the NIH.

The “one step approach” is based on research done by HAPO and recommends a 2 h glucose tolerance test after an overnight fast. Upon arrival at the hospital, the patient’s fasting blood glucose level is tested. After this, a 75 g glucose load is administered and the 1 and 2 h plasma glucose levels are recorded [6]. This screening method is the same criteria for both pregnant and nonpregnant individuals and is endorsed by the ADA [322]. Any women whose glucose values are greater than 92 mg/dL (5.1 mmol/L) fasting blood glucose, 180 mg/dL (10.0 mmol/L) at one hour, or 153 mg/dL (8.5 mmol/L) at two hours are diagnosed with GDM [179]. The IADPSG criteria were implemented in 2013 and consist of these more stringent blood glucose values. For this reason, the prevalence in countries that use these criteria has increased significantly. An 8-year study following 136,705 women found that the prevalence of GDM increased from 8.5% to 14.7% based on the new criteria [323].

The “two step approach” is still recommended by ACOG and the NIH due to a lack of evidence about the impact of the “one step approach” on pregnancy outcome. Initial screening involves a 50 g oral glucose challenge test (GCT) and measures blood glucose concentration after 1 h. If this test is abnormal (values greater than 140 mg/dL), a second, 3 h, 100 g glucose challenge is recommended later [111]. According to ACOG, fasting blood glucose greater than 95 mg/dl (5.3 mmol/L), 1 h glucose greater than 180 mg/dl (10.0 mmol/L), 2 h glucose greater than 155 mg/dL (8.6 mmol/L), or 3 h glucose over 140 mg/dL (7.8 mmol/L) are indicative of a GDM diagnosis [179].

It is important to note that HbA1c is not a good measure to assess risk of GDM when compared to the protocols above because this glycemic marker tends to be lower in pregnant women, leading to underestimation of glycemic profile [324].

### 7.2. Challenges in Identifying Individual Risk for T1D Post-GDM

A major challenge in identifying individual risk for T1D post-GDM is the low rate of follow-up testing. Current guidelines recommend that patients complete a repeat oral glucose test between 6 and 12 weeks after delivery. Unfortunately, a notable percentage of patients fail to complete this testing, making them unavailable for follow-up care [325]. This post-delivery follow-up is crucial, as it is where the majority of patients at risk for autoimmune diabetes are tested for autoantibodies. This is because there is little evidence to support the universal screening for antibodies after a patient has been diagnosed with GDM as several studies report no difference in maternal–fetal outcomes between women with or without autoimmune GDM [65]. The primary risk during pregnancy lies in the glucotoxicity from hyperglycemia, rather than the autoantibodies. With this understanding, testing all pregnant women with GDM for autoimmunity may expose them to unjustified stress during an already taxing period [67]. Instead, it is recommended to select high risk patients such as young, slim women with a history of thyroid disease presenting with GDM for this type of testing post-delivery [63]. There is also a significant delay in disease progression for women who recover from GDM post-delivery. The average time it takes a patient to develop diabetes postpartum is 1.9 ± 1.0 years for T1D and 5.9 ± 4.8 years T2D [63]. For this reason, proper patient education is imperative when talking about risks postpartum.

### 7.3. Advances in Diagnostic Tools and Predictive Modeling

Currently, there is limited support for universal autoantibody testing in women with GDM. Clinicians are, however, gaining a better understanding of the maternal factors that increase the risk of autoimmunity, with the goal of reducing the misdiagnosis of T2D in this population [326]. In addition to age and BMI, women who require exogenous insulin during pregnancy or have a history of autoimmune conditions should be closely monitored in the years following delivery [327].

With regard to testing protocols, there is now greater clarity on which autoantibodies to screen for in GDM patients and the optimal timing for such testing. Women whose glucose levels remain impaired 6 to 12 weeks postpartum should undergo comprehensive testing for diabetes-related autoantibodies, HbA1c, fasting glucose, and a 75 g OGTT within the first year postpartum [326]. It is also important to note that the diagnostic threshold for LADA following GDM is lower than for typical T1D due to the slower progression of the autoimmune response [65].

## 8. Management Strategies

### 8.1. Antenatal Management of GDM

The primary goal of antenatal management of GDM is control of hyperglycemia. This involves a multifaceted approach, with dietary modifications and nutritional management playing a pivotal role. Nutrition therapy for GDM focuses on balancing macronutrients to maintain stable blood glucose levels while ensuring adequate nutritional intake for both the mother and the developing fetus. This typically involves a diet similar to the general diabetes diet which is low in simple sugars and high in fiber, with careful monitoring of carbohydrate intake. One main difference between GDM and general diabetes is that the mother must still consume enough carbohydrates to support a growing fetus. The Institute of Medicine (IOM) recommends a diet with at least 175 g of carbohydrates daily [35]. Registered dietitians often work closely with pregnant women to create personalized meal plans that cater to individual caloric and nutritional needs, while also considering food preferences and lifestyle. The goal is to achieve glycemic control through a balanced diet, which can significantly reduce the need for medication.

Physical activity is another crucial component in the management of GDM. Regular exercise during pregnancy can help improve insulin sensitivity and lower blood glucose levels. However, it is important to tailor exercise recommendations to each woman’s pre-pregnancy activity level, medical history, and the progression of pregnancy. Healthcare providers often advise monitoring physical exertion and avoiding high-impact or high-risk activities. Exercise plans should be developed in consultation with healthcare professionals to ensure safety for both the mother and the baby [328].

If glucose levels remain elevated after 1–2 weeks of lifestyle interventions, and fetal growth is above the 75th percentile, pharmacologic treatment should be initiated [35]. Traditionally, exogenous insulin has been the primary glycemic treatment because it is effective and safe to use with a fetus as it does not cross the placenta. When starting treatment, 0.3 IU/kg body weight can be started every 24 h and be increased up to 1 IU/kg if needed. In addition to insulin, metformin can be administered as an oral agent. Metformin works by suppressing hepatic glucose production that leads to a reduction in fasting glucose. While this medication is a first line therapy for T2D patients, metformin can cause gastrointestinal symptoms, low vitamin B12, and does cross the placenta. In randomized clinical trials, metformin treatment was comparable to insulin in immediate neonatal outcomes [329]. Other medications are generally not indicated for fear of unexpected fetal complications [35].

The advent of Continuous Glucose Monitoring (CGM) has revolutionized diabetes care, offering a new horizon for care in GDM. Emerging research advocates for the integration of CGM in the care regimen for women with GDM, highlighting its potential to not only reduce average blood glucose levels but also diminish maternal weight gain and infant birth weights compared to the traditional blood glucose monitoring (BGM) methods. However, current evidence is limited and there is still a critical need for more extensive clinical trials.

### 8.2. Therapeutic Options and Long-Term Outlook Post-GDM

For the majority of patients, glucose regulation typically normalizes after delivery, allowing for the discontinuation of insulin and oral hypoglycemic agents. However, maintaining lifestyle modifications is strongly recommended [179]. In rare cases, hyperglycemia may persist postpartum, in which case insulin or glyburide may be prescribed, as both are considered safe for use during breastfeeding without adverse effects on the infant [330]. Patients with autoimmune GDM are at increased risk of experiencing a return of glucose intolerance within 1 to 5 years after delivery. This condition is often classified as LADA and should be managed accordingly [65]. Unlike T1D, LADA does not typically present with rapid weight loss or ketoacidosis. Instead, β-cell failure progresses more slowly, and patients often exhibit symptoms similar to those of T2D, including polyuria, polydipsia, polyphagia, and visual disturbances. The key factors for diagnosing LADA are the presence of autoantibodies, adult onset of the disease, and the absence of ketoacidosis [331].

### 8.3. Role of Insulin Therapy in Preventing Diabetes Progression

Early insulin therapy is crucial for achieving optimal glycemic control and preventing complications, such as ketoacidosis [332]. While exogenous insulin is fundamental in managing hyperglycemia in patients with active T1D, its potential to halt or slow the disease progression is still not clear. Insulin is a well-established target of the autoimmune response in T1D, and it has been hypothesized that early insulin administration may delay disease onset by allowing β-cells to rest and reducing T-cell infiltration into the pancreatic islets [333]. However, clinical studies evaluating this hypothesis have yielded mixed results. In trials involving relatives of T1D patients with a greater than 50% risk of developing the disease within five years, participants were administered low doses of parenteral or oral insulin and compared to placebo groups [334,335]. Unfortunately, neither study demonstrated a significant delay in disease progression compared to placebo [333]. In contrast, preclinical animal models have shown that insulin therapy can delay or even prevent T1D onset, suggesting potential mechanisms that have yet to be fully replicated in human trials [336].

In the context of GDM and T2D, insulin therapy has produced mixed outcomes. A study of 599 women with GDM revealed that while insulin treatment reduced birth weight, it did not lower the risk of developing diabetes at either 3 or 12 months postpartum [320]. This finding indicates that although exogenous insulin provides significant benefits for both mother and fetus during pregnancy, such as reducing the risk of macrosomia, it does not appear to confer long-term protection against progression to T2D [320]. Another study observed that even in women with mild GDM, insulin therapy during pregnancy did not reduce the likelihood of developing obesity or metabolic syndrome 5 to 10 years post-delivery [337].

### 8.4. Emerging Pharmacological Therapies for GDM

Glucagon-like peptide-1 receptor (GLP-1R) agonists are increasingly recognized for their multifaceted role in improving glucose regulation, reducing appetite, and promoting weight loss, which are all critical aspects of managing GDM [338,339,340]. These agents act by mimicking the effects of the incretin hormone GLP-1, which is naturally secreted by the gut in response to food intake [341]. By binding to GLP-1 receptors, they enhance glucose-dependent insulin secretion while simultaneously suppressing glucagon release, thus reducing hepatic glucose output [342]. This dual mechanism helps to mitigate the postprandial glucose spikes that are commonly observed in GDM, where insulin resistance and β-cell dysfunction are key pathophysiological features.

In addition to their glycemic effects, GLP-1R agonists improve insulin sensitivity, particularly in peripheral tissues like skeletal muscle and adipose tissue [343]. This can reduce the compensatory demand on pancreatic β-cells, alleviating the risk of further β-cell dysfunction during pregnancy. Moreover, GLP-1R agonists exert effects on appetite regulation by acting on hypothalamic pathways that control satiety [344]. Their ability to reduce caloric intake and promote weight loss is particularly valuable for obese or overweight women with GDM, as obesity significantly exacerbates insulin resistance and the risk of adverse pregnancy outcomes.

Beyond their metabolic effects, emerging evidence suggests that GLP-1R agonists may exert anti-inflammatory properties [345,346,347]. Chronic low-grade inflammation, driven by elevated levels of pro-inflammatory cytokines such as TNF-α and IL-6, is a hallmark of GDM. By modulating inflammatory pathways, GLP-1R agonists may help to mitigate this aspect of GDM pathophysiology, potentially improving both maternal and fetal outcomes. These anti-inflammatory effects may also play a role in reducing the long-term risk of progression to T2D and cardiovascular complications, which are more common in women with a history of GDM.

Despite these benefits, the use of GLP-1R agonists during pregnancy requires cautious evaluation due to concerns about their impact on fetal development. Early studies suggest that these agents do not cross the placenta in significant amounts, which could limit direct fetal exposure. However, comprehensive clinical trials are necessary to confirm their safety profile, as data on their long-term effects on fetal growth and development remain limited. Additionally, the potential for these drugs to influence placental function and maternal–fetal glucose transfer warrants further investigation.

The therapeutic potential of GLP-1R agonists in GDM management represents a paradigm shift, particularly for women who struggle to achieve glycemic control through lifestyle interventions alone. These agents could offer an alternative or adjunct to traditional pharmacological treatments, such as insulin, which remains the gold standard but poses challenges due to its invasive administration and risk of hypoglycemia. Moreover, their role in preconception care for women at high risk of GDM is an area of active research, as weight loss and improved insulin sensitivity before pregnancy may reduce the incidence of GDM altogether.

Future studies should focus on assessing the efficacy and safety of GLP-1R agonists in large, diverse populations of pregnant women. Additionally, research into potential combination therapies that include GLP-1R agonists alongside traditional treatments could provide insights into optimizing glycemic control while minimizing adverse effects. If proven safe and effective, GLP-1R agonists could become a cornerstone in the pharmacological management of GDM, improving outcomes for both mothers and their offspring.

## 9. Long-Term Follow-Up and Outcomes

### 9.1. Tracking Outcomes of Individuals with a History of GDM

Individuals with a history of GDM encounter a significantly elevated risk of developing both diabetes and CVD later in life. The likelihood of these adverse outcomes is influenced by several factors, including the severity of GDM, whether the condition is autoimmune in origin, and the patient’s capacity to adopt lifestyle modifications to prevent disease progression through regular follow-up care. Women who manage their glycemic control through lifestyle interventions alone, and who also breastfeed, demonstrate a lower risk for developing CVD and T2D compared to those who require pharmacological intervention [348]. Importantly, nearly all women with a history of GDM are estimated to have a ten-fold greater risk of developing diabetes compared to the general population [326]. Approximately 50% of women with GDM will develop some form of impaired glucose metabolism within a decade of diagnosis [265]. A 2020 follow-up study of 457 women with mild GDM, both treated and untreated, revealed that one-third of the cohort developed metabolic syndrome within seven years. Thes findings suggest that any degree of glucose intolerance during pregnancy (whether meeting GDM criteria or not) substantially increases the risk of metabolic disorders [349].

Beyond T2D, GDM predisposes women to an increased risk of obesity, hypertension, and dyslipidemia, all of which are key contributors to the development of ischemic heart disease [350]. A recent population-based study from Canada further demonstrated that even women with elevated blood glucose levels during pregnancy, who did not meet the formal diagnostic criteria for GDM, had a significantly increased risk of future CVD [351]. This evidence supports the growing advocacy for treating GDM as a pre-cardiovascular disease state, aiming to mitigate future risks of glycemic and cardiac dysfunction [350].

In cases of autoimmune GDM, the postpartum period is often more reflective of the patient’s autoimmune status than during pregnancy, as the immunosuppressive effects of pregnancy wane, allowing autoimmune antibody levels to rise. Studies have shown that overt diabetes typically manifests within 1 to 5 years postpartum in women with autoimmune GDM, with approximately 20% developing the condition within the first year following pregnancy [352].

Current evidence indicates that GDM most commonly manifests in women aged 25 to 35 years, with increasing prevalence in women over 30 years, reflecting trends in delayed childbearing [25,26,27,353]. Advanced maternal age, often defined as 35 years and older, is a well-established risk factor due to age-related declines in beta-cell function and the compounding effects of insulin resistance. Younger women, particularly those under 25 years, are less commonly affected but remain at risk when additional factors, such as obesity, polycystic ovary syndrome, or family history of diabetes, are present. Longitudinal data should stratify age-related trends in GDM incidence to better understand the interaction between age, pregnancy-related metabolic stress, and longer-term risks, such as progression to T2D, enabling targeted prevention and intervention strategies.

### 9.2. Identifying Biomarkers for T1D Progression

The identification of autoantibodies is a well-established approach for detecting biomarkers associated with T1D progression. that also has significant implications for GDM. Autoantibodies associated with T1D could potentially serve as early indicators of β-cell stress or autoimmunity in GDM, particularly in women with genetic predispositions or a family history of autoimmune conditions. Exploring the presence and role of these autoantibodies in GDM patients may help identify those at higher risk of postpartum T1D progression or latent autoimmune diabetes. Genetic biomarkers, such as HLA-DR/-DQ alleles, strongly associated with T1D risk, also provide a compelling opportunity to assess β-cell vulnerability in GDM. [253]. The presence of high-risk genotypes such as HLA-DR3/4 and HLA-DQ8 in GDM patients could indicate shared genetic pathways contributing to β-cell dysfunction [354]. This information could help stratify GDM patients into subgroups with varying levels of risk for T1D development, enabling closer monitoring and tailored interventions.

Emerging research on T-cell biomarkers further bridges the understanding of immune mechanisms in T1D and GDM. As primary mediators of β-cell destruction in T1D, T-cells represent a highly specific target for biomarker discovery [16,355]. Investigating T-cell activation profiles in GDM could uncover immune signatures that either precede or overlap with the autoimmune processes in T1D. These findings may illuminate the transition from GDM to T1D in at-risk individuals, particularly postpartum women who are vulnerable to autoimmune activation. Integrating T-cell biomarkers into longitudinal studies of GDM patients could significantly enhance risk stratification and inform early therapeutic strategies aimed at preserving β-cell function [356].

By leveraging the insights gained from biomarker research in T1D, advancements can be extended to GDM, fostering a unified approach to understanding and managing these interconnected conditions. Incorporating such biomarkers into clinical practice has the potential to bridge diagnostic and therapeutic gaps, providing a foundation for earlier intervention and improved outcomes in both GDM and T1D.

### 9.3. Challenges in Long-Term Follow-Up Studies

One of the major challenges in postpartum care for GDM is the high rate of patient loss to follow-up. Due to the latency period between childbirth and the onset of diabetes-related symptoms, which can range from 0 to 10 years, many patients discontinue regular medical visits once their symptoms resolve, and blood glucose levels return to normal. This disengagement significantly elevates the risk of developing severe complications, including metabolic syndrome and cardiovascular disease. These findings underscore the critical need for improved patient education, ensuring that women are fully aware of their increased long-term risk for developing chronic conditions [65]. A 2015 study identified fragmented care, inadequate information provision, and a lack of risk perception as key factors contributing to low follow-up adherence among women with GDM [348]. Additionally, poor coordination between hospital departments often leads to delays in testing and results, further increasing patient frustration. The study also highlighted that adopting a patient-centered approach and fostering trusting relationships between patients and healthcare providers could enhance follow-up rates. By emphasizing the importance of ongoing monitoring for postpartum complications, even in the absence of symptoms, such strategies could improve long-term outcomes [348].

## 10. Conclusions and Future Directions

GDM represents a pivotal challenge in maternal and neonatal health, with implications that extend far beyond pregnancy. Its increasing prevalence necessitates a proactive approach that integrates early detection, personalized management strategies, and long-term monitoring. Advances in molecular research, genetics, and immunology continue to unravel the complex pathways linking GDM to future metabolic disorders, offering new avenues for intervention. While lifestyle modifications remain the cornerstone of management, emerging pharmacological therapies and technological innovations may refine treatment approaches. Addressing disparities in screening and follow-up care is crucial to mitigating the intergenerational impact of GDM. A multidisciplinary focus on prevention, patient education, and precision medicine will be essential in reducing the burden of GDM and improving maternal–fetal outcomes.

Despite significant strides in understanding GDM, there remain critical knowledge gaps that demand further investigation. Future research should prioritize early biomarker discovery to enable precise risk stratification before pregnancy, allowing for targeted preventive measures rather than reactive management. A deeper exploration of metabolomic and proteomic profiles could provide novel insights into GDM progression and identify previously unrecognized molecular pathways contributing to insulin resistance and β-cell dysfunction. Additionally, neuroendocrine mechanisms linking maternal metabolic health to fetal brain development remain an underexplored area. The potential role of maternal hyperglycemia in influencing fetal cognitive outcomes, emotional regulation, and neurodevelopmental disorders warrants extensive longitudinal studies. Investigating how placental exosome signaling mediates fetal programming could reveal key intervention points to disrupt the cycle of metabolic disease transmission.

From a clinical standpoint, integrating machine learning algorithms and artificial intelligence (AI) into prenatal care could revolutionize GDM screening and management. Predictive analytics leveraging multi-omics data, wearable biosensors, and patient-reported metrics could enable dynamic risk assessment and real-time decision-making. Such innovations may transform prenatal care from a one-size-fits-all model to a precision-driven, adaptive approach tailored to individual risk profiles. Furthermore, while dietary and exercise interventions remain fundamental, their effectiveness varies among individuals due to genetic, microbiome, and metabolic differences. The emerging field of nutrigenomics and microbiome-based therapies offers a promising frontier in GDM management, potentially allowing for tailored dietary recommendations based on an individual’s gut microbiota composition and genetic predisposition to glucose intolerance. Probiotic and prebiotic interventions may emerge as adjunctive therapies to modulate maternal glucose metabolism and mitigate the inflammatory milieu associated with GDM.

Addressing psychosocial and behavioral determinants of GDM management is another critical future direction. Given the high postpartum attrition rates in diabetes screening and lifestyle adherence, behavioral economics and digital health solutions such as gamification, virtual coaching, and community-based peer support networks may help sustain long-term engagement in diabetes prevention strategies. Expanding culturally tailored interventions and leveraging mobile health applications with AI-driven chatbots could further enhance accessibility and adherence to recommended care plans. Furthermore, intergenerational prevention strategies should become a major focus in GDM research. Exploring how maternal hyperglycemia epigenetically influences fetal metabolic health could pave the way for transgenerational interventions that begin in early childhood or even preconception [357]. Pediatric monitoring of offspring exposed to GDM pregnancies, coupled with early metabolic and lifestyle interventions, may play a crucial role in curbing the rising tide of obesity and T2D in future generations.

By embracing these novel research directions, the field can evolve beyond conventional GDM management toward a comprehensive, predictive, and preventive care model that not only improves maternal health but also safeguards long-term offspring well-being.

## Figures and Tables

**Table 1:** Single-nucleotide polymorphisms (SNPs) linked to gestational diabetes mellitus (GDM) development.

SNP	Gene	Function	Affected Population
rs780094	Glucokinase regulator (*GCKR*) gene	Glycogen and triglyceride synthesis	Western Pacific and South America
rs2237895	Potassium voltage-gated (*KCNQ1*) gene	Calcium efflux from the β cells	South Korea and Middle East
rs1387153	Melatonin 1B receptor gene (*MTNR1B*)	Regulatory process of insulin secretion, glucose metabolism, and heart rate	European, North American, and Western Pacific populations
rs10830963	Melatonin 1B receptor gene (*MTNR1B*)	Regulatory process of insulin secretion, glucose metabolism, and heart rate	European, North American, and Western Pacific populations
rs1501299 rs2241766	Adiponectin gene (*ADIPOQ*)	Adipose tissue and adipokine release	Saudi Arabia
rs7903146	Transcription factor 7-like 2 (T*CF7L2*) gene	Impacts insulin secretion and β-cell function via the Wnt signaling pathway	European and Asian populations
rs7756992	Cyclin-dependent kinase 5 regulatory subunit-associated protein 1-like 1 (*CDKAL1*) gene	Influences β-cell function and insulin secretion	Multiple ethnic groups
rs4402960	Insulin-like growth factor 2 mRNA-binding protein 2 (*IGF2BP2*) gene	Involved in insulin secretion and β-cell function	Asian populations

**Table 2:** Genes associated with gestational diabetes mellitus (GDM).

Gene	Chromosome Location	Associated Risk	Function	References
*TCF7L2*	10q25.3	Increased risk of type 2 diabetes	Most strongly associated with type 2 diabetes risk	[150,151]
*GCK*	7p13	Altered glucose metabolism	Plays a crucial role in pancreatic beta-cell function	[135]
*MTNR1B*	11q21	Disrupted melatonin signaling	Melatonin receptor affecting insulin secretion	[152,153]
*CDKAL1*	6p22.3	Impaired beta-cell function	Associated with reduced insulin release	[122,154]
*IGF2BP2*	3q27.2	Regulation of insulin production	Influences insulin-mediated glucose homeostasis	[159,160]
*KCNQ1*	11p15.5	Impaired insulin secretion	Encodes the pore-forming potassium (K+) channel alpha-subunit	[155,156]
*IRS1*	2q36.3	Insulin resistance and impaired insulin secretion	Plays a critical role in the insulin-signaling pathway	[157,158]
